# Falls leading to fracture and nutrition among older adults: a case–control study

**DOI:** 10.1186/s41043-023-00361-x

**Published:** 2023-03-13

**Authors:** Mahdieh Ardaneh, Mohammad Fararouei, Jafar Hassanzadeh

**Affiliations:** Shiraz University of Medical Sciences, Shiraz, Islamic Republic of Iran

**Keywords:** Fall, Older adults, Physical activity, Nutrition

## Abstract

**Objectives:**

Injurious falls, especially those leading to bone fracture, are major causes of death and disability among older people. Our aim was to measure the association of nutritional factors and physical activity with falls leading to fracture among Iranian older adults.

**Methods:**

This is the second phase of a previously published case–control study on 300 patients and 590 controls.

**Results:**

In addition to the socio-economic factors that were reported before, our results revealed that consumption of fish, vegetables, fruits, and nuts reduced the risk of falling, whereas consumption of cheese, red meat, and sweets raised the risk of falls among the participants.

**Conclusion:**

The results of our study suggested that diets rich in fish meat fruits and vegetables should be encouraged in the everyday life of older adults. We suggest health officials to take these important results into consideration when planning protective measures.

## Introduction

With the global expanding population of older adults, and the increase in the incidence of chronic diseases, it is vitally important that health care authorities identify and understand factors that affect health and quality of life in this growing part of the population [1]. There are numerous concerns about the health hazards and determinants of quality of life of older adults. However, despite the wide variation of the health diminishing conditions among older adults, the prevention strategies are divided into only three categories: physically active lifestyle, healthy nutrition and cognitive stimulation [2].

Statistics suggest that accidents (with falling as the most common cause of accident) are the fifth cause of death among older people [3]. In addition, falling comprise the cause of %80 of elderlies’ admission to hospitals due to accident-related injuries and the rate of mortality of falling-related injuries among older adults is 2.7% [4]. Moreover, about %22 to %60 of falls cause injuries, of which about 10–15% result in serious conditions such as fractures and head trauma [5]. With regard to falls-related factors, the results of a study showed that, via increasing physical fitness, the level of physical activity may have a preventive effect on falls among older people [6]. Another study suggested that malnutrition is associated with higher mortality in older adults who experience hip fracture [7]. Nutrition is also revealed to be important in all stages of life as it has fundamental effects on individual’s health, especially in older ages. Rath et al. suggested that a good way to sustain optimal level of health during entire life is to eat a balanced diet which caters the specific nutritive needs of the older adults [8]. On the other hand, poor nutrition is associated with frailty in older people. As a result, screening for early diagnosis of malnutrition and effective interventions are essential in the correction of macro- and micronutrient deficiencies among older adults [9, 10]. This is why many researchers believe that dietary interventions and physical exercise may have positive impacts on muscle mass and muscle function and reduce the risk of falling [11].

In Iran, an older adult is defined as a person who is aged equal or more than 60 years. According to the results of a census carried out in 2017, 7.5 million people (equivalent to about 9.3% of the total population of Iran) are individuals who have reached their 60 s. It is estimated that in a decade, %10.7 of the Iranian population will be older adults [12, 13].

Despite the importance of the possible effects of nutrition on the risk of accidents among the aging population, no study investigated this important association in Iran. This study aimed to identify the effect of nutrition on falls leading to fracture among Iranian older adults. The results will help us in understanding the nutritional predisposing factors of injuries among older adults. The results of our study will also contribute to the better and more effective falls preventive measures among older adults.

## Material and methods

This is the second phase of a case–control study conducted from September 2018 to May 2019 in Shiraz, the capital of Fars province. In this study, all patients over 60 who were admitted to one of the two city’s referral orthopedic centers (Chamran and Ragaei hospitals) due to falling-related fractures were invited to participate. As the largest orthopedic centers, the hospitals deliver medical cares to patients from the Southern part of the country. Details of the study setting and methods are provided before [14].

### Selection of cases

Patients were between 60 to 100 years old and were admitted to the above-mentioned hospitals during the study period due to falling-related bone fractures (at least one fracture). Patients were actively registered with a family physician, were mentally and physically able to take part in the interview and were able to walk (not in a wheelchair or in bed) before the falling occurred.

### Selection of controls

Almost all Iranian population are registered with the national health system and receive health services from a health center. Using the database of the system, we selected a random sample of individuals with the distribution of health center, gender and age (± 5 years) similar to the case group. A call was made to selected controls to be invited to a face-to-face interview in their health center. All controls were able to walk (not on a wheelchair or in bed), were between 60 and 100 years old, and were able to take part in the interview. A trained health nurse conducted the interview after arranging the meeting via the phone call.

Exclusion criteria: Patients were excluded if they were not able to read numbers from 0 to 10 and were not well enough to consciously answer the interviewer’s questions. Patients in ICU were not selected because they were not able to fully cooperate in the interview.

### Data collection

To collect the required data, an interview administered questionnaire was designed by a team of two epidemiologists, a public health nurse, and a physician. To define the reliability of the questionnaire, we conducted a pilot study on 20 participants who were interviewed twice in a two-week interval [15]. Based on the results of a test–retest analysis, we believe the questionnaire was adequately reliable (Cronbach's alpha = 0.75). A trained health nurse interviewed the case and control participants during a daily visit to the hospitals or health centers. Accordingly, 304 patients and 608 controls agreed to be faced to face interviewed. Due to the illiteracy of a significant number of participants, verbal consent was obtained from all but 5 eligible patients (due to feeling not well enough to answer the questions) and 8 controls (were too busy to come to the health centers). Of those invited to the study, 4 patients (0/01%) and 18 controls (0/03%) refused to participate in the study.

The collected data covered the demographic status of the participants (i.e., sex, age, current and previous jobs, education, living with a spouse, adequacy of income, and the number of children), lifestyle (i.e., physical activity, duration of physical activity, type of physical activity, quality of Life, shopping outdoors, who makes food, and hobby) and health status (i.e., weight and height (BMI). As the second phase of the interview, a second questionnaire that was previously designed and evaluated with the same procedures as the first one used to define the nutritional habits of the participants. In that regard, the usual consumption of red meat (portion per week, p/w), chicken meat (p/w), fish (p/w), pickles (p/w), sweets (p/w), type of oil consumed (saturated or unsaturated), vegetables (p/w) and fruits (p/w), and dairy products (p/w) were reported by the participants [16]. Tea and coffee were reported on a daily base (cups/day), but the rest of the food groups were reported as portions per week. The required demographic and lifestyle information was self-reported by the respondents.

The participants were assured that their information is used for research proposes only.

Sample size and statistical analysis: Based on the results of the pilot study, the required sample size for the first phase of the study was defined and accordingly, 304 patients and 608 controls were selected. For the second phase, a post hoc power analysis suggested that the selected sample size was adequate to find a significant association between the consumption of red meat and risk of falls leading to fracture as (OR as small as 1.5, with a power of > 80%).

The qualitative and quantitative variables are reported as frequency, proportions, means, and standard deviations as adequate. We used student t and chi-square tests to detect unadjusted associations between the study variables and falling. The adjusted associations were measured by refitting the final logistic model from the phase one. We included variables with significant effects on the fitness of the model. To do the analysis, SPSS version 22 and R (version 4.0.2) was used (the significance level was set at *P* < 0.05).

## Results

In total, 300 patients with injurious falls, and 590 healthy individuals participated in this case–control study [14]. Previously we reported the associations of falling with socio-economic, health and lifestyle factors (Tables 1, 2). Here, we focus on the results of the analysis on the nutritional factors.

**Table 1:** Distribution of quantitative and qualitative variables regarding demographic and lifestyle factors in case and control groups

Variable	Category/scale	Cases (300) Mean ± SD or *N* (%)	Controls (590) Mean ± SD or *N* (%)	*P* value*
Age	Years	72.20 ± 11.30	71.10 ± 6.60	0.13
Gender	Male	136 (46.10)	273 (46.10)	0.83
	Female	164 (53.90)	317 (53.90)	
Education	Illiterate	172 (57.30)	89 (15.10)	< 0.001
	Primary and Secondary	91 (30.00)	295 (50.00)	
	Diploma ≤	38 (12.70)	206 (34.90)	
Occupation	Self-employed	108 (36.0)	185 (31.40)	< 0.001
	Employee	37 (12.30)	172 (29.20)	
	Housewife or unemployed	155 (51.70)	233 (39.50)	
Currently employed	Yes	80 (26.70)	63 (10.70)	< 0.001
Working under the sun	Yes	66 (22.00)	98 (16.70)	0.032
Income	Inadequate	183 (61.00)	203 (34.50)	< 0.001
	Adequate and good	117 (39.00)	386 (65.50)	
Type of accommodation	House	257 (85.70)	480 (81.40)	0.11
	Apartment	43 (14.30)	110 (18.60)	
Hobby	TV	138 (46.00)	320 (54.30)	< 0.001
	Chatting Family	66 (22.00)	100 (17.00)	
	Chatting Friends	21 (7.00)	93 (15.80)	
	Nothing	75 (25.00)	77 (12.90)	
Use of footwear	Yes	75 (25.00)	257 (43.60)	< 0.001
Sleep hours per night	Hours	7.00 ± 2.30	7.30 ± 1.80	0.001
Sleep hours per day	Hours	0.50 ± 1.00	0.70 ± 0.80	0.003
Sleep quality	Score (0–10)	7.90 ± 2.80	8.40 ± 2.10	0.01
Physical activity	Yes	146 (48.70)	373 (63.20)	< 0.001
Type of physical activity	Exercise	116 (38.70)	322 (54.60)	< 0.001
	Work	50 (16.70)	102 (17.30)	
	No physical activity	134 (44.70)	166 (28.10)	
Smoking	Yes	121 (40.30)	132 (22.40)	< 0.001

^*^P values are based on Chi-Square or independent samples t test

**Table 2:** Distribution of quantitative and qualitative variables regarding health-related factors in case and control groups

Variable	Category/scale	Case (300) Mean ± SD or *N* (%)	Control (590) Mean ± SD or *N* (%)	*P* value*
BMI	Kg/m^2^	23.90 ± 4.30	25.30 ± 3.90	< 0.001
Perceived vision impairment	Yes	163 (54.30)	274 (46.40)	0.03
Wearing glasses	Yes	84 (28.00)	251 (42.50)	< 0.001
Hearing problem	Yes	62 (20.70)	84 (14.20)	0.01
Vertigo	Yes	128 (42.70)	124 (21.00)	< 0.001
Knee pain	Yes	179 (59.70)	288 (48.80)	0.002
Backache	Yes	116 (38.70)	244 (41.40)	0.44
Walking with aid	Yes	107 (35.70)	112 (19.00)	< 0.001
Fall in the past 5 years	Yes	94 (31.30)	86 (14.60)	< 0.001

*P values are based on Chi-Square or independent samples t test

Based on the results of bivariate analysis, diet and particular foods are associated with injurious falling (*P* < 0.05 for all) (Table 3).

**Table 3:** Nutritional status of case and control groups

Variable	Category/Scale	Cases *n* (%) Mean ± SD or *N* (%)	Controls *n* (%) Mean ± SD or *N* (%)	*P* value*
Red meat	Portions/week	0.96 ± 0.97	0.94 ± 0.86	0.76
Fish	Portions/week	0.38 ± 0.60	0.84 ± 0.77	< 0.001
White meat	Portions/week	1.86 ± 1.10	2.01 ± 1.03	0.06
Egg	Portions/week	2.03 ± 1.52	2.21 ± 1.41	0.07
Milk	Portions/week	1.85 ± 1.76	2.43 ± 1.85	< 0.001
Yogurt	Portions/week	2.08 ± 1.63	2.42 ± 1.54	0.003
Dugh	Portions/week	1.57 ± 1.67	2.08 ± 1.62	< 0.001
Cheese	Portions/week	2.88 ± 1.68	2.85 ± 1.70	0.80
Tea	Cups/day	3.60 ± 2.70	3.60 ± 2.30	0.78
Coffee	Cups/day	0.09 ± 0.29	0.15 ± 0.48	0.05
Fruits	Occasionally	162 (54.00)	246 (42.70)	< 0.001
	Often	138 (46.00)	344 (58.30)	
Vegetables	Occasionally	166 (55.30)	192 (32.50)	< 0.001
	Often	134 (44.70)	398 (67.50)	
Sweets	Not at all	66 (22.00)	197 (33.40)	< 0.001
	Occasionally	234 (78.00)	393 (66.60)	
Nuts	Not at all	92 (30.70)	44 (7.50)	< 0.001
	Occasionally	208 (69.30)	547 (92.50)	

*P values are based on Chi-Square or independent samples t test

Based on the results of multiple logistic regression, strong associations between the risk of injurious fall and several health-related and nutritional factors were observed (Table 4): Accordingly, inverse association between BMI (OR_kgr/m_^2^ = 0.94, 95% CI 0.89–0.98, *P* = 0.009), physical activity (OR_no/yes_ = 0.64, 95% CI 0.42–0.96, *P* = 0.31), consumption of fish (OR_portions/week_ = 0.48, 95% CI 0.35–0.65, *P* =  < 0.001), dugh (OR_cups/week_ = 0.77, 95% CI 0.66–0.88, *P* =  < 0.001), vegetables (OR_often/ not at all_ = 0.36, 95% CI 0.14–0.88, *P* < 0.001), and nuts (OR _often/ not at all_ = 0.18, 95% CI 0.10–0.31, *P* =  < 0.001) with falls were observed. On the other hand, smoking (OR_no/yes_ = 1.83, 95% CI 1.20–2.71, *P* = 0.005), consumption of red meat (OR_portions/week_ = 1.52, 95% CI 1.21–1.91, *P* =  < 0.001), cheese (OR_portions/week_ = 1.24, 95% CI 1.09–1.42, *P* = 0.002), and sweets (OR_often/not at all_ = 2.86, 95% CI 1.77–4.63, *P* =  < 0.001) were directly associated with injurious falling.

**Table 4:** The association of study variables with the risk of falling among the participants

Variable	Category/scale	OR	95% CI	*P* value
BMI	Kg/m^2^	0.94	0.89–0.98	0.009
Hobby	TV	Ref	–	< 0.001
	Chatting family	1.67	0.99–2.82	
	Chatting friends	0.24	0.12–0.49	
	Nothing	1.58	0.93–2.69	
Currently employed	No	Ref	–	< 0.001
	Yes	6.69	3.88–11.50	–
Education	Illiterate	Ref	–	< 0.001
	Primary and secondary	0.18	0.11–0.28	–
	Diploma ≤	0.10	0.05–0.17	
Physical activity	No	Ref	–	0.031
	Yes	0.64	0.42–0.96	–
Smoking	No	Ref	–	0.005
	Yes	1.83	1.20–2.71	–
Red meat	Portions/week	1.52	1.21–1.91	< 0.001
fish	Portions/week	0.48	0.35–0.65	< 0.001
Cheese	Portions/week	1.24	1.09–1.42	0.002
Dugh	Cups/week	0.77	0.66–0.88	< 0.001
Vegetables	Not at all	Ref	–	< 0.001
	Occasionally	0.49	0.32–0.74	–
	Often	0.36	0.14–0.88	
Sweets	Not at all	Ref	–	< 0.001
	Often	2.86	1.77–4.63	–
Nuts	Not at all	Ref	–	< 0.001
	Often	0.18	0.10–0.31	–

## Discussion

This study was conducted to identify the association of nutritional factors with the risk of falling that lead to fracture among all older individuals who were admitted to the two orthopedic referral centers in Shiraz during the study period. The first phase of the present study suggested that BMI, hobby, currently working, education, income, sleep quality, vertigo and smoking affect the risk of falling among older adults. The current study revealed that consumption of fish meat, vegetables, nuts, and tea are inversely associated with the risk of falling-related fractures. On the other hand, consumption of cheese red meat, and sweets were directly associated with the risk of falling-related fractures.

Regarding the nutritional factors, the results of the current study revealed that the risk of fall was inversely associated with BMI. This finding is in accordance with the results of a study conducted by O'Neill et al., who suggested that being underweight is a risk factor for falling among older adults [17]. Similarly, Mazur et al., suggested that higher BMI is associated with a lower risk of falling among older adults [18]. The index may affect the chance of falling via strengthening the muscles and bones and therefore reduce the risk of injurious falls.

According to our results, elderly people who are not physically active are more prone to injurious falls. It seems that exercise in older age prevents the accident via promoting health, body function and muscle strength. Older people who exercise are also usually more physically active in their younger and middle age life. A meta-analysis of randomized controlled trials suggested that exercise in older people is effective in preventing fall-related fractures among older adults [19].

In addition, our study revealed that diets reach in fish meat, low fat dairy products (e.g., watery yogurt), fiber, and tea may prevent falls leading to fractures in older adults. Bakhtiari et al. suggested that malnutrition in older people can lead to serious health concerns, including muscle weakness and bone-loss followed by an increase in falls and higher risks of hospitalization and death [20]. In addition, Shibasaki et al. suggested that one of the most effective ways of preventing frailty among older adults is to keep appropriate diet. In addition, Trevisan et al. suggested that malnourished subjects experience higher risk of fall compared to those who were well-nourished [21]. In that regard, researchers suggested that the consumption of red meat, fish, soybeans, milk, fruit and vegetables decreases the prevalence of frailty [22]. Moreover, Laird et al. suggested that more yogurt consumption is associated with the increased bone mineral density and physical function in older adults [23]. It has been shown that drinking caffeinated tea and coffee prevent drowsiness, resulting in fewer falls in older ages. Shen et al. in a study in China, suggested that traditional lifestyle of drinking tea could be a cost-effective way toward healthy aging [24].

Our study showed that consumption of sweets, cheese and red meat may increase the risk of falls leading to fractures in the older adults. Movassagh et al. suggested that consumption of soft drinks, fried foods, red meat, processed food products, sweets, and desserts are harmful to the health of bones [25]. However, Rogers et al. suggested that western diet (including foods like hamburgers, fries and processed meats, cheese, sweets, and desserts) has direct relationship with bone density in older men [26]. These controversies may represent different routes of action of our diet on falls leading to fracture. For example, although western food may have positive effects on bone density, they may, on the other hand, cause more frailty, vertigo, and dizziness due to higher blood pressure and hyperlipidemia [27].

## Strength and limitations

The study participants are selected from a relatively representative sample of the older people in the community. However, the study focused on older people whose falling resulted in severe injuries, requiring hospitalization. As a result, our sample is only a small part of a much wider population of older people who fall. In addition, the design of our study prevents us from making confident causal inference about the findings.

## Conclusion

Policymakers counteracting the epidemic of falls are to implement educational programs to improve the lifestyle (particularly diet) of older people. The two phases of our study suggested that physical exercise, social life, and appropriate diet should be supervised in the life of older adults. We suggest family physicians, nurses, and health officials to apply these important suggestions in their everyday work.

## Data Availability

The datasets generated and/or analyzed during the current study are not publicly available due to its being the intellectual property of Shiraz University of Medical Sciences but are available from the corresponding author on reasonable request.

## Abbreviations

OR: Odds ratio
CI: Confidence interval
BMI: Body mass index

## Declarations

### Patient and public involvement

Patients and the public were not involved in this research.
